# Osteoblastoma and Osteoid Osteoma of the Mandible: Review of the Literature and Report of Two Cases

**DOI:** 10.1155/2022/7623855

**Published:** 2022-03-08

**Authors:** Taylor Ellingsen, Andrew Nalley, Dolphine Oda, Thomas B. Dodson, Peggy P. Lee

**Affiliations:** ^1^School of Dentistry, University of Washington, Box 356370, 1959 NE Pacific St, Seattle, WA 98195, USA; ^2^Clinical Instructor, Division of Radiology, Department of Oral Medicine, School of Dentistry, University of Washington, Box 356370, 1959 NE Pacific St, Seattle, WA 98195, USA; ^3^Department of Oral and Maxillofacial Surgery, School of Dentistry, University of Washington, Box 357131, 1959 NE Pacific St, Seattle, WA 98195, USA; ^4^Department of Oral Medicine, School of Dentistry, University of Washington, Box 356370, 1959 NE Pacific St, Seattle, WA 98195, USA

## Abstract

Osteoblastoma and osteoid osteoma are rare benign neoplasms of the jaws. We reviewed current literature surrounding the ongoing debate over similarities and differences of osteoblastoma and osteoid osteoma and present two cases. Both cases are well-demarcated mixed radiodensity mandibular lesions with histological features of osteoblastoma. They exhibit, however, distinctly unique and contrasting clinical and imaging characteristics suggesting that the first case is osteoblastoma and the second is osteoid osteoma. The first case of a 37-year-old male presents with a large, expansile lesion at posterior mandible, surrounded by a thick sclerotic band. Unusual features include significant buccal/lingual expansion, extensive new bone apposition, and soft tissue edema in the masseter muscle. This is in contrast to the second case of a much smaller lesion in a 17-year-old male with history of recent third molar extraction in the left posterior mandible. In this case, CT imaging revealed a circular, nonexpansile lesion with a sclerotic border surrounded by a radiolucent rim. Both patients underwent surgical excision of the lesion with extraction of the adjacent tooth. We discuss herein the distinct clinical and imaging features.

## 1. Introduction

Osteoblastoma and osteoid osteoma are benign and slow-growing neoplasms of the bone with similar, if not identical, histopathological features. Some have proposed that any benign neoplasm of the maxilla or mandible that is composed of osteoblasts forming osteoid and bone trabeculae set in a well-vascularized connective tissue stroma be classified as a single entity, osteoblastoma [1, 2]. However, the World Health Organization (WHO) continues to classify them as two separate entities [3, 4]. Osteoid osteoma was initially described by Heine in 1927 with Jaffe the first to recognize it as a unique entity in 1935 [5]. Osteoblastoma was first described in1932 by Jaffe and Mayer as “an osteoblastic-osteoid tissue forming tumor” [6].

Osteoblastoma is a rare tumor of the bone, accounting for approximately 3% of all benign bone tumors and 1% of primary bone tumors [7]. Osteoblastoma is most commonly found in the spine or areas of cancellous bone [8]. It is rare in the maxillofacial region with only 10% of osteoblastomas occurring in the skull [9]. When they do occur in the maxillofacial region, there is a marked predilection for the mandible, specifically the posterior mandible [7]. Osteoblastoma has a reported 2 : 1 male to female ratio; however, there is no gender predilection in the summary report of 77 cases in maxilla or mandible [2]. They are more common in younger patients, generally presenting in the second through fourth decades of life with a mean age of 24 [2, 10, 11].

Osteoid osteomas are more common than osteoblastomas accounting for approximately 10-12% of all benign bone tumors and 3% of primary bone tumors [7]. These lesions are more common in the lower extremity long bones, where more than 50% of lesions are reported to occur [7]. Osteoid osteoma, by contrast, is commonly located within cortical bone [8]. Like osteoblastoma, these lesions are exceptionally rare in the maxillofacial region with a tendency for the mandible when they do occur [10, 12, 13]. Osteoid osteoma has a characteristic nocturnal pain pattern and limited growth potential usually presenting small in size. Gender predilection and average age range in the maxillofacial region are similar to that of osteoblastoma [2, 10, 12].

Radiographically, both lesions usually appear as well-circumscribed, mixed density lesions surrounded by a sclerotic rim. Osteoid osteoma usually displays a larger area of sclerotic bone formation than osteoblastoma [7, 10]. Osteoblastoma is known to expand and erode the surrounding bone. In osteoid osteoma, a discrete central area of lucency known as the nidus with patchy mineralization and an ovoid shape can be appreciated [7, 10, 12]. This radiographic presentation of the central nidus is most easily appreciated in cortical lesions and may be more difficult to distinguish if the lesion arises from medullary bone [7].

Histologically, osteoblastoma and osteoid osteoma appear similarly and are characterized by the abnormal proliferation of osteoblasts forming osteoid and woven bone superimposed on a well-vascularized fibrous connective tissue stroma [1, 7, 10]. Additionally, immunohistochemical staining was similar in both osteoid osteoma and osteoblastoma [1]. Others believe that there are subtle histological and immunological differences between the two bony neoplasms such as the presence of epithelioid osteoblasts in more aggressive types of osteoblastoma, the presence of S-100 protein in osteoid osteoma, and the increased COX-2 expression in osteoid osteoma [10, 14].

We report herein the details of two cases with histological features of osteoblastoma with distinct clinical and imaging characteristics. We will review case reports of osteoblastoma and osteoid osteoma in the maxilla and mandible. The literature search was conducted using PubMed and Google Scholar search. The search term for osteoblastoma literature was “osteoblastoma AND mandible OR jaw”. The search term for osteoid osteoma literature was “osteoid osteoma AND mandible OR jaw”. We will further discuss the ongoing debate over the similarities and differences between osteoblastoma and osteoid osteoma.

## 2. Case Presentation

### 2.1. Case #1

#### 2.1.1. Clinical Presentation

A 37-year-old male presented to the clinic with pain in the left posterior mandible and preauricular area that had been progressively worsening over the past four months. Pain was managed with nonsteroidal anti-inflammatory drugs (NSAID) and acetaminophen at the time of initial exam.

Clinically, there were no changes to occlusion or paresthesias. The range of motion was within normal limits. Both intraoral and extraoral exam demonstrated swelling in the ascending ramus area of the left mandible with pain on palpation. All four wisdom teeth are impacted and not visible clinically. Probing depths were less than 4 mm in the lower left quadrant.

Past medical history is significant for treatment of a mandibular fracture 11 years prior to presentation with no complications but is otherwise noncontributory.

#### 2.1.2. Imaging Features

**Figure 1:**
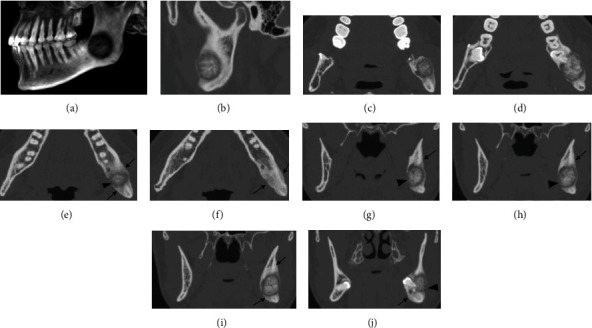
Case #1 3D CT reconstruction (a) and bone window CT sagittal view (b) showing the mixed density lesion associated with impacted #17 measuring 1.6 × 2.1 × 2 cm. The border of the lesion is well circumscribed with a broad sclerotic rim surrounding a lucent halo. Axial views ((c-f) from superior to inferior) and coronal views ((g-j) from posterior to anterior) showing buccal and lingual expansion, thinning, and areas of perforation at buccal and lingual cortices (arrowheads). Extensive sclerotic reactive bone is seen surrounding the lesion. New bone formation can be seen at buccal, lingual cortical, and inferior to the lesion (arrows). The lucent rim of the lesion extends into the left mandibular canal, with potential involvement of the inferior alveolar nerve (g–j).

**Figure 2:**
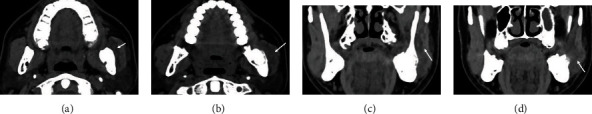
Case #1, soft tissue window CT images. Axial views (a–b) and coronal views (c–d) showing edema within the adjacent left masseter muscle (arrows).

Medical grade CT revealed a well-defined, mixed radiodensity lesion within the body of left mandible, measuring 1.6 × 2.1 × 2.0 cm. The lesion is located in the left posterior mandibular angle, involving the root of full bony impacted #17 and extending to the inferior third of the mandibular ramus. The internal structure was mixed but predominantly densely mineralized with a surrounding lucent halo. Buccal and lingual expansion, thinning, and areas of perforation at buccal and lingual cortices can be appreciated. There was a broad zone of reactive sclerotic bone surrounding this lesion (Figure 1). Large periosteal bone formation can be seen at buccal, lingual cortical, and inferior aspects of the lesion. The lucent rim of the lesion extends into the left mandibular canal, with potential involvement of the inferior alveolar nerve (Figure 1). Edema was noted within the adjacent masseter muscle (Figure 2).

#### 2.1.3. Differential Diagnosis

Due to size and radiographic presentation, the differential diagnosis for this lesion in the left posterior mandible includes osteoblastoma, osteosarcoma, osteoid osteoma, ossifying fibroma, focal cemento-osseous dysplasia or calcifying odontogenic cyst with odontoma. Radiographically, osteosarcoma usually presents with poorly delineated borders, making an osteosarcoma diagnosis less likely for the lesion in question. Given the large size of the lesion, a diagnosis of osteoid osteoma would be unlikely. Ossifying fibroma does not typically present with a wide zone of sclerotic bone formation as seen in this lesion. Due to the expansile nature of the lesion, focal cemento-osseous dysplasia is unlikely in this case.

### 2.2. Case #2

#### 2.2.1. Clinical Presentation

**Figure 3:**
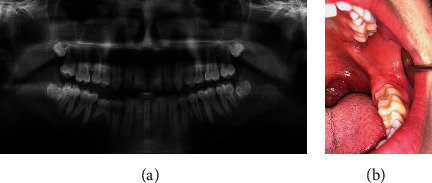
(a) Panoramic radiograph of case #2 prior to third molar extraction showing partially developed and bony impacted #1, 16, 17, and 32. No signs of pathology noted around the impacted #17. (b) Clinical photo post extraction with noted scar tissue at #17 extraction site but otherwise no abnormalities or swelling.

A 17-year-old male was referred to an oral surgeon for surgical extraction of complete bony impacted third molars (Figure 3(a)). For his third molar extractions, a full thickness mucoperiosteal flap was opened with crestal bone removal and buccal trough for delivery. The sockets were irrigated, and the surgeon reported no direct visualization of any adjacent structures. 4-0 chromic sutures were placed, and gauze packs were applied over sockets to obtain hemostasis. Eight days later, the patient returned for a post-op appointment where healing was within normal limits and pain levels were minimal. Three months after the initial follow-up appointment, the patient started feeling pain in the area of #17 that progressively worsened over the next 5 months.

**Figure 4:**
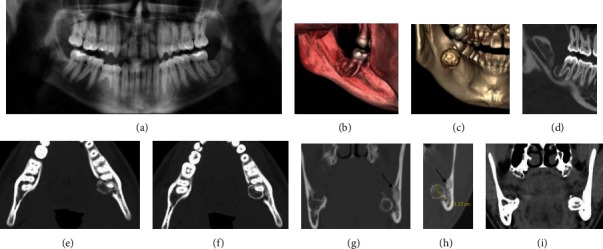
(a) Case #2 panoramic radiograph. (b and c) 3D CT reconstruction, viewed from the lingual aspect. (d) CBCT sagittal view. (e–f) Bone window CT axial views. (g–h) Bone window CT coronal views. (i) Soft tissue window CT coronal view. Panoramic radiograph was taken 8 months postsurgical extraction of the impacted tooth #17. An exophytic mixed density lesion, measuring 1.2 × 1.0 × 1.1 cm, emanated from the distal root of tooth #18. The border of the lesion is well-demarcated with a sclerotic rim surrounded by a lucent rim. Small area of bony sclerosis is noted at buccal and superior of the lesion (arrows) (g and h). There was no evidence of root resorption at associated root of #18. Unlike case 1, there is no evidence of expansion, new bone formation, or adjacent soft tissue edema (i).

Panoramic and CBCT imaging revealed a small well-circumscribed lesion distal and lingual of tooth #18 (Figures 4(a) and 4(d)). The oral surgeon did a surgical exploration of the area to obtain a biopsy of the tissue. The initial biopsy returned as fibrous tissue with retained foreign materials, stating that there was a “golden-brown pigmented material with lipid vacuoles likely represents an intrasocket medication.”

The patient initially said he was feeling better after the surgical exploration, but by the next month, the pain had returned and was continually worsening. The patient was then referred to the Oral and Maxillofacial Surgery Department at the University of Washington for further evaluation and treatment. Upon exam at the University of Washington, the patient was found to have radiating pain to his ear. The patient described it as “shooting nerve pain that gets worse at night.” Patient reported intermittent vague swelling, but no bad taste. At this time, he was controlling the pain with ibuprofen. Intraoral examination revealed scar tissue at extraction site but no swelling, erythema, drainage, or abnormally appearing tissue (Figure 3(b)).

#### 2.2.2. Imaging Features

Panoramic imaging 8 months post extraction when the patient began feeling pain in the area revealed a small mixed density lesion associated with the distal root of #18 which was not present in the panoramic image prior to #17 extraction (Figures 3(a) and 4(a)).

Cone beam CT image obtained by the oral surgeon prior to initial biopsy revealed a well-defined, mixed density lesion with a soft tissue capsule surrounding a cortical border on the distal and lingual aspects of #18. The lesion has eroded and expanded beyond the lingual cortical plate (Figure 4). No root resorption associated with #18 distal root was appreciated.

A medical CT scan was obtained several months after the CBCT to evaluate the changes from the CBCT as well as potential soft tissue involvement. Imaging revealed a 1.2 × 1.0 × 1.1 cm lesion emanated from the distal root of #18 that had eroded and expanded beyond the lingual cortical plate. A small area of bony sclerosis is noted at the buccal and superior aspects of the lesion. The lesion appeared similarly on bone window CT as the previous CBCT. There was no soft tissue involvement (Figure 4).

#### 2.2.3. Differential Diagnosis

Given the radiographic findings and characteristic pain presentation, osteoid osteoma was considered to be high on the differential diagnosis followed by osteoblastoma. Close relation the distal root of #18 added cementoblastoma to the differential. However, the distal root of #18 showed no evidence of root resorption making this diagnosis unlikely.

## 3. Treatment and Histopathology

**Figure 5:**
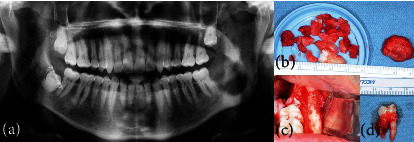
(a) Case #1 panoramic radiograph one month post excisional biopsy showing the lesion and #17 completely removed with bony sclerosis still visible. (b) Case #1 surgical specimen. (c) Case #1 intraoperative clinical photo. (d) Case #2 surgical specimen showing the lesion in association with the distal root of #18.

Case #1 was treated with enucleation and full bony extraction of #17. The lesion was removed in pieces (Figures 5(b) and 5(c)). Cauterization was used to obtain hemostasis and the defect was closed using running 3-0 chromic sutures.

Panoramic radiograph obtained one month post excisional biopsy demonstrated complete removal of the lesion and #17 with evidence of bony sclerosis still visible (Figure 5(a)).

Case #2 was treated with enucleation, curettage, and extraction of #18. The site was irrigated and closed using multiple interrupted 3-0 chromic sutures. Adequate hemostasis was obtained with primary closure. Figure 5(d) depicts the surgical specimen, showing the lesion in association with the distal root of #18.

**Figure 6:**
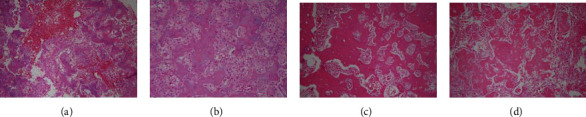
Histological features of case #1 (a and b). (a) Decalcified bone with evident reversal lines with prominent osteoblastic activity. Note the vascular connective tissue stroma with dilated blood vessels and hemorrhage (H&E stain, ×40). (b) A higher magnification of panel (a) showing the bone with prominent and large osteoblasts with abundant cytoplasm and epithelioid morphology (H&E stain, ×100). (c and d) Histological features of case #2. (c) Decalcified bone with reversal lines with prominent osteoblastic activity. Note the vascular connective tissue stroma with dilated blood vessels and small foci of hemorrhage (H&E stain, ×40). (d) A higher magnification of panel (c) showing the bone with prominent and large osteoblasts with abundant cytoplasm and vascular connective tissue stroma with dilated blood vessels and scattered erythrocytes (H&E stain, ×100).

Histopathological interpretation of both biopsy samples obtained from excisional biopsy was consistent with osteoblastoma. Biopsy specimens were represented by multiple fragments of decalcified hard and soft tissue made up of bone at different stages of development (Figures 6(a), 6(c), and 6(d)), some mineralized with other areas made up of new bone formation. The mineralized bone showed reversal lines. All bone fragments showed prominent osteoblastic activity with abundant cytoplasm in some areas (Figures 6(a), 6(b), and 6(d)). Scattered osteoclastic activity was noted. The connective tissue stroma in both specimens was loose and vascular. It contained many dilated blood vessels and foci of hemorrhage (Figures 6(a), 6(b), and 6(d)).

## 4. Discussion and Literature Review

Though both cases presented were diagnosed as osteoblastoma histopathologically, they differ significantly in both clinical presentation and imaging characteristics. Taken together, case #1 is considered osteoblastoma and case #2 is considered osteoid osteoma (Table 1). It has been reported that clinical presentation, size, location, and imaging features are the most important factors in determining the definitive diagnosis of these two similar lesions [7]. The clinical and radiographic features of reported osteoblastoma and osteoid osteoma cases not included in the previous reviews by Jones et al. and An et al. are presented in Tables 2 and 3 [2, 12].  Table 4 summarizes the characteristics of all cases reviewed here (see Table 2 and Table 3), those reviewed by Jones et al., An et al., and our two new cases.

Clinically, both osteoblastoma and osteoid osteoma commonly present with pain; however, the pain pattern is specific for each neoplasm. Pain reported in osteoblastoma is described as dull, aching, and progressively worsening over time. It is thought to be caused by local expansion of the tumor and does not typically respond to treatment with NSAIDs [15]. A typical pain presentation of osteoid osteoma, seen in around 80% of patients, involves localized pain which worsens at night and is usually greatly relieved by the use of NSAIDs [2, 14]. The unique pain pattern of osteoid osteoma can be explained by an abundance of nerve fibers in the nidus matrix, elevated levels of prostaglandins, and increased expression of COX-2. These features are not found in osteoblastoma or other benign osseous lesions [7, 14]. The pain presentation of case #2 was consistent with a classic osteoid osteoma presentation endorsing worsening pain at night that was well controlled with NSAIDs. In our review, nocturnal pain was only reported in two of the 11 osteoid osteoma cases and one of the 31 osteoblastoma cases. The reason for the low incidence of nocturnal pain in case reports maybe due to underreporting by the clinicians or patients, which underscores the limitation of reviews of published case reports.

Another important difference between the two lesions is size. Osteoblastoma typically presents as >1.5-2 cm in diameter and is known for its tendency to cause bony expansion [7, 8]. The average size of osteoblastoma in our review was 2.97 cm (Table 4). Case #1 fits these criteria measuring 2.1 cm at its greatest dimension. Osteoid osteoma has a more limited growth potential, usually presenting smaller than 1.5-2 cm with the nidus generally <1 cm [7]. The average size of osteoid osteomas in our review was 1.16 cm (Table 4). Case #2 fits these criteria measuring at 1 cm in diameter.

The average age of patients in our review diagnosed with osteoid osteoma was similar but slightly older than those diagnosed with osteoblastoma with averages of 26.63 years and 22.76 years, respectively. This is consistent with previous reports of these lesions predominantly arising in the first and second decades of life [2, 10, 11]; however, rare case reports included older patients as well (Table 3). Male to female ratios were close to 1 : 1 for both lesion types, differing from WHO classification which state a 2 : 1 male to female ratio [3, 4]. It is possible that the gender predilection for these lesions is different for the maxillofacial region than it is for the rest of the body.

Radiographically, most osteoblastoma and osteoid osteoma cases reviewed in Table 4 were described as mixed density. Lesions diagnosed as osteoblastoma were more likely to present as predominantly radiolucent than lesions diagnosed as osteoid osteoma. 25 osteoblastoma cases were reported as predominantly radiolucent compared to only two lesions diagnosed as osteoid osteoma. Lesions diagnosed as osteoid osteoma were more likely to have a surrounding area of sclerosis. As summarized in Table 4, sclerosis was seen in 15/33 (45%), of osteoid osteoma cases compared to only 3/97 (3%) of osteoblastoma cases. These findings are similar to previous reports [7, 10]. Of the lesions reviewed in Tables 2 and 3, 6 osteoblastoma and 4 osteoid osteoma cases were found to have an area of surrounding radiolucency. Cases reviewed by An et al. and Jones et al. did not include this information and therefore cannot be compared.

Case #1 demonstrated a unique degree of soft tissue edema in the adjacent masseter muscle as well as the extensive formation of new, reactive bone (Figures 1(c)–1(f) and 2). To the best of our knowledge, this is the first report of muscle edema and extensive new bone formation associated with osteoblastoma. These unusual features cannot be related to the aggressiveness of the tumor as histologic analysis did not indicate aggressive osteoblastoma. It is possible that soft tissue edema has been underreported in the past due to the choice of image modality.

The lesion in case #2 developed shortly after routine third molar extractions. While history of extraction has been reported in other cases of osteoblastoma and osteoid osteoma, the timing of this presentation is closer to time of extraction, occurring only 8 months after the procedure [13, 16]. It is possible that this lesion may have formed in response to third molar extraction or the associated healing process; however, there is a lack of evidence to justify this hypothesis. Other osteoblastoma cases, including case #1, have a history of nonspecific facial trauma which again may or may not be associated with the formation their lesions [11]. Many cases reviewed in this paper did not state whether or not there was a history of trauma. Given the anecdotal association, further investigation is required to determine if trauma is a contributing factor to the formation of these lesions.

With so few reports of these benign bony neoplasms available in the literature, misdiagnoses can be common and preoperative diagnoses are often variable. In 24 cases of osteoblastoma, Jones et al. found 37.5% of preoperative diagnoses to be a fibro-osseous process such as fibrous dysplasia, ossifying fibroma, or focal osseous dysplasia, 25% to be a bone tumor or bone disease, 20.8% to be an odontogenic cyst, 12.5% to have no preoperative diagnosis, and 4.2% to be a salivary gland neoplasm [2]. This initial misdiagnosis can be seen in case #2 where foreign body reaction was suspected from initial biopsy results.

The recommended treatment for osteoblastoma and osteoid osteoma can be very different, thus highlighting the importance of distinguishing between the two lesions despite similar histopathological features. Our reviews here as well as previous case reports support that osteoid osteoma and osteoblastoma can and should be distinguished from one another based on reported features [3, 4, 17]. For osteoid osteoma, nonsurgical management with NSAIDs is an option as it can effectively relieve pain in some cases [7]. There are few studies on the long-term effectiveness of nonsurgical management. Some studies have indicated that conservative treatments can be as effective as surgical management [18]. Some reports have even claimed that lesions can spontaneously regress while being treated nonsurgically with NSAIDs [19]. Osteoblastoma on the other hand is always treated surgically due to a lack of responsiveness to nonsurgical pain management and potential for aggressive behavior [20]. Though the relationship is not well understood, many have noted similarities between aggressive type osteoblastoma and osteosarcoma [1, 5, 21]. This relationship contributes to the urgency seen in treatment methods of osteoblastoma as compared to those of osteoid osteoma.

Recurrences of both lesions have been described in the literature. It has been found that osteoid osteomas recur slightly less often than osteoblastomas, with a recurrence rate of 4.5% compared to 9.8%, respectively [8]. Additionally, osteoblastoma may have other closely related lesions such as atypical sclerosing osteoblastic neoplasm that have an even higher recurrence rate of about 10-21% [14, 20]. To further complicate the relationship, there have been several reports of osteoid osteoma transforming into or recurring as osteoblastoma [1, 7, 22]. Some believe that osteoid osteoma is simply an immature osteoblastoma that happened to be discovered earlier in its developmental course. To the best of our knowledge, both cases #1 and #2 remain disease free today.

While the WHO still classifies osteoblastoma and osteoid osteoma as separate tumors, it has been recommended by some to reclassify these two neoplasms as single and separate disease processes [1–4]. Others suggest that the nomenclature should be changed to reflect distinct clinical presentations of the same pathological process [8]. Still more, some believe that the current classification should remain, keeping the neoplasms as separate entities. Further investigation of the benign bony lesions including clinical and radiographic presentation, location, size, demographics, history of trauma, and behavior is needed in order to better understand the relationship between osteoblastoma and osteoid osteoma. A better understanding of this relationship will aid in the diagnosis and management of these lesions in the maxillofacial region. In this paper, we shared two cases of osteoblastoma. They each had unique imaging and clinical characteristics as well as some previously unreported attributes. Along with the existing literature, these cases can contribute to the ongoing research, treatment, and debate behind these lesions.

## Figures and Tables

**Table 1:** Characteristics of osteoblastoma and osteoid osteoma as previously summarized [7, 8, 10] comparing to current cases.

	Sex	Chief complaint	Imaging features	Size (cm)
Osteoblastoma [7, 8, 10]	M ≥ F	Mild to moderate pain. Less relief from aspirin (unlike osteoid osteoma).	Mixed to radiopaque, variable. Surrounded by sclerotic bone. Expansile, erosive. Arises in medullary bone.	≥1.5-2
Case #1	M	Progressively worsening pain and swelling. Pain managed by NSAID.	Mostly radiopaque mass. Surrounded by sclerotic bone. Expansile, erosive. Arises in medullary bone.	2.1
Osteoid osteoma [7, 8, 10]	M ≥ F	Moderate to severe pain. Progressive pain worsening at night and responds well to NSAIDs.	Lucent or mixed nidus. Surrounded by sclerotic bone. Arises in cortical bone.	<1.5-2
Case #2	M	Progressively worsening pain beginning 8 months post surgical extraction. Shooting pain worse at night and controlled with ibuprofen.	Lucent nidus with patchy mineralization. Arises in lingual cortex.	1

**Table 2:** Characteristics of previously reported osteoblastoma of the maxilla and mandible (*n* = 29).

Case	Author (year)	Age	Sex	Chief complaint	Site	Imaging features	Size (cm)
1	Yamada (2009) [23]	29	M	Pain, swelling	Maxilla, hard palate	Mixed density mass with narrow radiolucent zone	2
2^∗^	Lin (2012) [24]	10	M	Pain, swelling	Mandible, anterior	Radiopaque mass with an irregular border and an ill-defined margin	4 × 3
3	Lin (2012) [24]	26	F	Pain	Mandible, anterior	Expansile, mixed radiolucent, and radiopaque lesion with a radiolucent rim	.5 × .5
4	Bokhari (2012) [25]	18	M	Swelling, slight pain	Maxilla	Well-circumscribed, radiopaque mixed with areas of radiolucency. Surrounded by a well-defined radiolucent rim. There was no reactive bone forming rim	3 × 2
5	Pérez (2012) [26]	7	F	Painless facial asymmetry	Mandible, body to condyle	Well-defined multilocular mass with honeycomb areas	NS
6	Rawal (2006) [27]	30	F	Pain	Mandible, body to parasymphysis	Well-defined radiolucency	2 × 2
7	Rawal (2006) [27]	31	F	Pain	Mandible, body	Well-defined radiolucency	2 × 2
8	Rawal (2006) [27]	16	M	Pain	Maxilla, canine to premolar	Well-defined radiolucency	5 × 4
9	Rawal (2006) [27]	29	F	Pain	Mandible, body	Poorly defined, mixed radiodensity	3 × 2
10^†^	Rawal (2006) [27]	18	F	Pain	Mandible, body	NS	5 × 5
11^†^	Rawal (2006) [27]	15	F	Pain	Mandible, body	Well-defined radiopaque	2 × 1 × 1
12^†^	Rawal (2006) [27]	78	M	Pain	Mandible, body	Soft tissue opacity overlying residual alveolus	2.5 × 1.75 × 1
13	Mahajan (2013) [28]	45	F	Swelling, lymphadenopathy	Mandible, posterior body	NS	5 × 3.5
14	More (2012) [16]	40	F	Swelling, hx of extraction	Mandible, posterior body	Well-defined compact trabecular pattern, with dense bone in certain areas of the lesion	4.5
15	Sheikh (2014) [29]	45	F	Pain and swelling	Mandible, posterior body	Mixed radiolucent-radiopaque lesion with sclerotic borders. Loss of trabeculation with normal surrounding bone	2 × 3.5
16	Shah (2013) [30]	7	M	Pain and swelling	Mandible, posterior body	Homogeneous radiopaque expansile. The adjacent tooth germs were displaced Recurrent: large well-defined mass composed of two locules with multiple internal calcifications	Original: 3.5 Recurrent: 2.5 × 2 × 1.5 and 3 × 3 × 1.5
17^∗^	Kaur (2012) [31]	26	F	Pain and swelling	Mandible, posterior body	Well-delineated expansile radiolucency contained calcified mass and few radiopaque flecks scattered within the radiolucency. Expansion and thinning of the lower border of the mandible	3 × 3
18^∗^	Castro (2016) [32]	7	F	Pain and swelling	Mandible, posterior body	Poorly defined mixed radiolucent-radiopaque	8
19^∗^	Vinuth (2013) [33]	25	M	Pain and swelling	Mandible, posterior body	Ill-defined radiolucency with internal radiodensities	5 × 4
20^∗^	Harrington (2011) [34]	25	M	Mild pain and swelling	Maxilla	Ill-defined radiolucency with internal radiodensities	4
21	Woźniak (2010) [35]	30	M	Swelling	Mandible, body	Poorly marginated from adjacent tissue. Small, irregular radiolucent foci of bone destruction with a few patchy calcifications are visible centrally	4 × 5
22	Angiero (2006) [36]	24	M	Swelling	Mandible, posterior body	Poorly defined, “ground-glass” radiopaque lesion	1
23	Angiero (2006) [36]	8	M	Swelling, missing teeth	Maxilla	Mixed pattern of radiolucency and radiopacity	1.5
24^∗∗^	Mardaleishvili (2014) [20]	12	F	Pain, swelling	Mandible, body	Well-defined, radiolucent with minimal calcification	3.5
25	Capodiferro (2005) [9]	16	F	Pain and swelling	Mandible, posterior body	Deformity of the bone architecture and containing large amounts of calcified material. The lesion is not associated with sclerotic borders or periosteal alteration	3
26	Capodiferro (2005) [9]	10	M	Pain and swelling	Mandible, body	NS	2.5
27	Capodiferro (2005) [9]	21	F	Pain and swelling	Mandible, body	NS	2
28	Capodiferro (2005) [9]	20	M	Pain and swelling	Mandible, posterior body	Radiolucent lesion of the left mandibular molar area, in close association with an unerupted tooth, with regular contours and containing fine calcifications	3
29	Capelozza (2005) [37]	8	M	Failure of eruption	Mandible, anterior	Ill-defined borders, displaying varied degrees of radiopacity, surrounded by a radiolucent halo	1 × 1

This summary does not include 67 osteoblastoma cases summarized by Jones et al. in 2006. ^∗^Aggressive osteoblastoma. ^†^Periosteal osteoblastoma. ^∗∗^Lesions described as predominantly RL with some calcifications were classified as RL. NS: not stated; hx: history.

**Table 3:** Characteristics of previously reported osteoid osteoma of the maxilla and mandible (n =12).

Case	Author (year)	Age	Sex	Chief complaint	Site	Imaging features	Size (cm)
1	Singh (2011) [13]	20	M	Radiating pain and swelling, hx of extraction	Mandible, posterior body	Well-defined radiopacity with a radiolucent rim showing a central radiopaque nidus surrounded by a radiolucent border	3.5
2	Mohammed (2013) [38]	20	NS	Pain and swelling, NSAIDs effective	Mandible, posterior body	Mixed radiopaque radiolucent lesion. The roots of the second premolar and the first molar appeared to be involved	3 × 2
3	Thopte (2018) [39]	21	M	Swelling, reduced mouth opening	Mandible, condyle	Solitary ill-defined homogeneous mixed radiopaque-radiolucency with a thin sclerotic border on the left mandibular condyle.	4.5 × 3
4	Matthies (2019) [40]	18	M	Pain, worse at night, response to NSAIDs	Mandible, posterior	Unclear tumor mass, radiopaque with lucent rim	.9x.8 × .5
5	Betz (2017) [15]	18	M	Swelling, slight pain	Mandible, posterior body	Well-defined, noncorticated borders and a surrounding radiolucency, internally radiodense material showed a laminated pattern, focal destruction of the cortical plate	1 × .6 × .6
6	Porto (2007) [41]	23	F	Severe pain nonresponsive to NSAIDs, limited opening	Mandible, condyle	Well-circumscribed and predominantly radiopaque	1.1 × .8
7	Infante-Cossio (2017) [42]	44	F	Mild pain worse at night, response to NSAIDs	Mandible, posterior body	Sclerotic lesion with a well delineated central calcified nidus surrounded by a radiolucent band and reactive sclerosis	1
8	Devathambi (2017) [43]	13	F	Dull progressive pain with response to NSAIDs	Mandible, posterior body and ramus	Well-defined radiopaque mass	1.4 × 1.5
9	Díaz-Rengifo (2019) [44]	69	F	Incidental finding	Maxilla	Well-delimited radiopaque mass	.5 × .8
10	Roscher (2018) [45]	21	M	Severe pain, swelling	Maxilla	Radiopaque lesion with a radiolucent core and peripheral reactive sclerosis	.5
11	Khaitan (2016) [46]	40	M	Pain, swelling, loose tooth, bleeding	Maxilla	Ill-defined homogenous periarticular radiolucency	2 × 1
12	Bajpai (2018) [47]	54	M	Pain, swelling	Mandible, posterior	Well-defined radiopaque w central radiolucency	NS

This summary does not include 21 osteoid osteoma cases summarized by An et al. in 2013. NS: not stated; hx: history.

**Table 4:** Summary of all reported cases of osteoblastoma and osteoid osteoma of the maxilla and mandible, including current cases.

		Osteoblastoma (*n* = 97)	Osteoid osteoma (*n* = 33)
Age, mean (range)		22.76 (3-78)	26.63 (4-77)
Male, female, NS		42, 55	16, 15, 2
Maxilla		22	7
Mandible	Posterior	56	19
	Anterior	12	1
	Condyle	7	6
Symptoms	Asymptomatic	7	3
	Pain	67	25
	Swelling	63	17
Size (cm), mean (range)		2.93 (0.5-5)	1.16 (0.4-4.5)
History of trauma	Yes	4	1
	NS	93	32
Radiographic description	Radiolucent	25	2
	Radiopaque	20	11
	Mixed	42	18
	Surrounding sclerosis	3	15
	NS	10	2

This summary excludes one case from An et al. in 2013 due to location on the temporal bone. NS: not stated.
